# Betaine Regulates the Production of Reactive Oxygen Species Through Wnt10b Signaling in the Liver of Zebrafish

**DOI:** 10.3389/fphys.2022.877178

**Published:** 2022-04-28

**Authors:** Ao Li, Yaqi Gu, Xiuzhen Zhang, Hairui Yu, Dongwu Liu, Qiuxiang Pang

**Affiliations:** ^1^ Anti-Aging & Regenerative Medicine Research Institution, School of Life Sciences and Medicine, Shandong University of Technology, Zibo, China; ^2^ College of Biological and Agricultural Engineering, Weifang Bioengineering Technology Research Center, Weifang University, Weifang, China; ^3^ School of Agricultural Engineering and Food Science, Shandong University of Technology, Zibo, China

**Keywords:** betaine, Wnt10b, β-catenin, reactive oxygen species, zebrafish

## Abstract

When fish are under oxidative stress, levels of reactive oxygen species (ROS) are chronically elevated, which play a crucial role in fish innate immunity. In the present study, the mechanism by which betaine regulates ROS production *via* Wnt10b/β-catenin signaling was investigated in zebrafish liver. Our results showed that betaine enrichment of diet at 0.1, 0.2 and 0.4 g/kg induced Wnt10b and β-catenin gene expression, but suppressed GSK-3β expression in zebrafish liver. In addition, the content of superoxide anion (O_2_^·−^), hydrogen peroxide (H_2_O_2_) and hydroxyl radical (·OH) was decreased by all of the experimental betaine treatments. However, betaine enrichment of diet at 0.1, 0.2 and 0.4 g/kg enhanced gene expression and activity of superoxide dismutase (SOD), glutathione peroxidase (GSH-PX) and catalase (CAT) in zebrafish liver. In addition, Wnt10b RNA was further interfered in zebrafish, and the results of Wnt10b RNAi indicated that Wnt10b plays a key role in regulating ROS production and antioxidant enzyme activity. In conclusion, betaine can inhibit ROS production in zebrafish liver through the Wnt10b/β-catenin signaling pathway.

## 1 Introduction

Due to various stress factors in the aquatic environment, fish are prone to stimulate inflammatory responses (Saurabh and Sahoo, 2008; Troncoso, et al., 2012). In addition, the antioxidant status is closely related to the innate immunity of fish species (Kuang, et al., 2012; Tort, et al., 2003). Generally speaking, the existence of various antioxidant enzymes, the generation and elimination of ROS in fish are in a state of dynamic equilibrium. However, if the homeostasis is disrupted, excess ROS can be produced in fish tissues. Excessive production of ROS is closely related to lipid peroxidation, cellular damage and protein oxidation (Cooke, et al., 2003; Martínez-Álvarez, et al., 2005; Maynard, et al., 2009). Furthermore, high levels of ROS exacerbate oxidative stress and induce the production of pro-inflammatory cytokines (Naik and Dixit, 2011). To protect animals from ROS-mediated damage, various antioxidant enzymes, such as superoxide dismutase (SOD), glutathione peroxidase (GSH-PX) and catalase (CAT), play key roles in regulating the balance of ROS levels (Martínez-Álvarez, et al., 2005).

The chemical structural formula of betaine is (CH_3_)_3_N^+^–CH_2_COO^−^, which is a naturally occurring metabolite in animals (Craig, 2004). In addition, betaine, a by-product of sugar beet processing, is used commercially as a feed additive in aquaculture fish feeds (Eklund, et al., 2005). Today, betaine is widely used as an attractant in aquaculture. Moreover, betaine plays an important role in regulating cellular osmotic pressure (Nakanishi, et al., 1990). Previously, betaine was observed to accumulate in mammalian renal medulla cells and chicken fibroblasts under osmotic stress, thereby functioning to regulate osmotic pressure and monitor water content (Bagnasco, et al., 1986; Eklund, et al., 2005; Kidd, et al., 1997; Petronini, et al., 1992). In addition, betaine reduces the osmotic pressure of Atlantic salmon and improves the ability to maintain osmotic balance (Virtanen, et al., 1989). Addition of betaine to fish feed significantly improves feed utilization, survival, and fish growth (Craig, 2004; Liang, et al., 2001; Luo, et al., 2011; Wu and Davis, 2005).

Cytoplasmic accumulation of β-catenin is a decisive event in cells in the canonical Wnt signaling pathway. In addition, the molecule GSK-3β is involved in the regulation of cytoplasmic β-catenin levels (Logan and Nusse, 2004; Veeman, et al., 2003; Zeng, et al., 1997). After the Wnt molecule binds to the receptor complex on the cell membrane, the activity of GSK-3β decreases and β-catenin accumulates, which further regulates the expression of target genes (Bhanot, et al., 1996; Molenaar, et al., 1996). In a previous study, it was observed that the Wnt/β-catenin signaling pathway is involved in the regulation of oxidative stress in MC3T3-E1 cells (Qi, et al., 2016).

Under oxidative stress, ROS production in animals is chronically increased, disrupting cellular metabolism and fish immunity (Lushchak, 2014). Previously, betaine has been shown to have antioxidant properties in a variety of animals (Doğru-Abbasoğlu, et al., 2018; Heidari, et al., 2018; Wen, et al., 2019). However, the regulatory mechanism of betaine on ROS production *via* Wnt10b signaling remains unknown in fish species. Previous studies have shown that betaine increases stress resistance, and the Wnt/β-catenin signaling pathway plays a role in regulating oxidative stress (Kim, et al., 2014; Li, et al., 2019; Qi, et al., 2016; Zhang, et al., 2018). It is interesting to examine whether betaine regulates ROS production in fish through the Wnt10b signaling pathway. The aim of this study was to investigate the mechanism by which betaine modulates ROS production *via* Wnt10b/β-catenin signaling in zebrafish liver.

## 2 Materials and Methods

### 2.1 Diet and Animals

Betaine was purchased from Sunwin Biotechnology Co., Ltd. (Weifang, China). Casein and gelatin were used as protein sources. Four isoenergetic and isonitrogenic diets containing 0, 0.1, 0.2, and 0.4 g/kg betaine were prepared according to Table 1. The ingredients were ground into powder then passed through a 120 μm sieve, and mixed with lard and linseed oil. Then, the appropriate amount of water (300 ml/kg dry ingredient) was added to the ingredients and blended with a tablet machine to form flakes. Finally, the flakes were dried in a ventilated oven at 40°C for 10 h, pulverized, sieved into small particles (200–300 μm), and stored at −20°C for later use.

**TABLE 1:** Composition and proximate analysis of the experimental diets.

Items	Betaine level (g/kg diet)			
	0	0.1	0.2	0.4
Ingredients (g/kg diet)				
Casein	420	420	420	420
Gelatin	105	105	105	105
Dextrin	190	190	190	190
Lard oil	83	83	83	83
Linseed oil	17	17	17	17
Cellulose	100	99.9	99.8	99.6
Sodium carboxymethylcellulose	20	20	20	20
Vitamin premix^a^	10	10	10	10
Mineral premix^b^	40	40	40	40
Ca_2_(H_2_PO_4_)_2_	10	10	10	10
Choline chloride	5	5	5	5
Betaine	0	0.1	0.2	0.4
Total	1,000	1,000	1,000	1,000
Proximate composition				
Moisture (g/kg diet)	97.4	98.2	96.6	98.1
Crude protein (g/kg diet)	480.6	481.2	482.6	482.4
Crude lipid (g/kg diet)	103.4	102.9	104.5	103.5
Crude ash (g/kg diet)	61.2	63.2	63.8	63.2

Notes.

a Vitamin premix contained (mg/g mixer) thiamin hydrochloride, 5 mg; riboflavin, 5 mg; calcium pantothenate, 10 mg; nicotic acid, 6.05 mg; L-ascorbyl-2-monophosphate-Mg, 3.95 mg; alpha-tocopherol acetate, 50 mg; pyridoxine hydrochloride, 4 mg; folic acid, 1.5 mg; inositol, 200 mg; menadione, 4 mg; retinyl acetate, 60 mg; biotin, 0.6 mg. All ingredients were diluted with alpha-cellulose to 1 g.

b Mineral premix contained (g/kg diet) calcium biphosphate, 13.58 g; calcium lactate, 32.7 g; FeSO_4_·6H_2_O, 2.97 g; magnesium sulfate, 13.7 g; potassium phosphate dibasic, 23.98 g; sodium biphosphate, 8.72 g; sodium chloride, 4.35 g; AlCl_3_·6H_2_O, 0.015 g; KI, 0.015 g; CuCl_2_, 0.01 g; MnSO_4_·H_2_O, 0.08 g; CoCl_2_·6H_2_O, 0.1 g; ZnSO_4_·7H_2_O, 0.3 g.

Male zebrafish (AB strain, ∼3.2 cm) were cultured in 3.0 L flow-through glass jars with circulating dechlorinated water at 28°C on a 14 h light:10 h dark cycle. The fish were then fed ad libitum twice a day at 8:00 and 18:00 with commercial feed obtained from Sanyou Landscaping Feed Technology Co., Ltd. (Beijing, China) for 2 weeks of acclimation. Two different experimental rearings were performed, the first involving betaine levels in the experimental diet and the second involving Wnt10b RNA interference.

### 2.2 Animals Treatments

First, experimental feeding on the four betaine level diets was performed as follows. 180 male zebrafish were assigned to 12 tanks (15 fish per tank) and each betaine level (0, 0.1, 0.2 and 0.4 g/kg betaine) was distributed into three tanks. The fish were fed ad libitum twice a day at 8:00 and 18:00. 6 weeks later, after zebrafish were anesthetized with 0.1 g/L MS 222, a liver sample was collected from five fish per tank for gene expression analysis. In addition, another liver sample was collected from five fish in each tank for biochemical indicator analysis. Three replicates were used for each betaine level treatment group.

### 2.3 Wnt10b RNA Interference

First, the Wnt10b gene (AY182171.1) was cloned according to the primers listed in Table 2. Then, double-stranded RNA (dsRNA) was synthesized using the primers listed in Table 2 according to the previous method (Arockiaraj, et al., 2014). Experimental rearing for Wnt10b RNA interference (Wnt10b RNAi) was performed as follows. After 2 weeks of acclimation, 90 male zebrafish (∼3.8 cm) were allocated to six tanks for Wnt10b RNAi. For the RNAi group (3 tanks), fish were intraperitoneally injected with 500 ng of dsRNA diluted in DEPEC-treated water, while fish in the control group (3 tanks) were intraperitoneally injected with 5 μl of DEPEC-treated water. Then, the two groups of zebrafish were fed ad libitum a diet containing 0.4 g/kg betaine at 8:00 and 18:00 every day. After 6 days, the fish were sampled for analysis as the betaine treatment experiments.

**TABLE 2:** PCR primers for Wnt10b RNA interference.

PCR primers	Forward (5′-3′)	Reverse (5′-3′)
ORF	CAA​TGA​CAT​CCT​CGG​CCT​GAA​G	TCA​CTT​GCA​CAC​ATT​AAC​CCA​CTC
dsRNA	GAT​CAC​TAA​TAC​GAC​TCA​CTA​TAG​GGG​AGA​CCA​GCG​CTG​GAA​CTG​CTC	GAT​CAC​TAA​TAC​GAC​TCA​CTA​TAG​GGC​CAC​TCT​GTT​ATT​ACG​AAT​CC

### 2.4 Assay for Liver Level of Reactive Oxygen Species and Antioxidant Enzyme Activity

Liver samples were homogenized and centrifuged at 5,000 ×g for 10 min at 4°C, and supernatants were collected for biochemical analysis. The supernatant ·OH, H_2_O_2_, O_2_^·−^ levels, and CAT, SOD, GSH-PX activity were measured using commercial kits purchased from Nanjing Jiancheng Bioengineering Institute (Nanjing, China). The protein concentration in the supernatant was detected at 595 nm according to the Bradford method (Bradford, 1976). SOD activity was measured at 550 nm by measuring inhibition of the reduction rate of cytochrome c, and CAT activity was measured by analyzing residual H_2_O_2_ absorbance at 405 nm. Activity of GSH-PX was detected by analyzing the rate of NADPH oxidation at 412 nm. In addition, since H_2_O_2_ can form a stable complex with ammonium molybdate, the content of H_2_O_2_ was detected at 405 nm. The production of ·OH was detected according to the production of hydrogen peroxide, and the production of O_2_^·−^ was measured at 550 nm by the xanthine oxidase method.

### 2.5 RNA Extraction and Real-Time Quantitative Polymerase Chain Reaction

The primer sequences for various target genes and reference gene (β-actin) were shown in Table 3. Subsequently, real-time PCR was performed in a quantitative thermal cycler (ROCHE, Lightcycler 480, Switzerland) using SYBR^®^ Premix Ex Taq™ II (Takara, Japan). The program for quantitative RT-PCR was as follows: 50°C for 2 min, 95°C for 10 min, then 40 cycles of 95°C for 15 s and 60°C for 1 min. Finally, the relative gene expression level was detected by 2^−ΔΔCT^ method (Livak and Schmittgen, 2001).

**TABLE 3:** Real-time quantitative PCR primers for genes of zebrafish.

Target gene	Forward (5′-3′)	Reverse (5′-3′)	Size (bp)	GenBank
Wnt10b	TCC​TGA​AAC​AGG​CTC​GAA​GT	GCT​GCT​CAC​TTG​CAC​ACA​TT	112	AY182171.1
GSK-3β	TCT​GCT​CAC​CGT​TTC​CTT​TC	CTC​CGA​CCC​ACT​TAA​CTC​CA	115	NM_131381.1
β-catenin	GGA​GCT​CAC​CAG​CTC​TCT​GT	TAG​CTT​GGG​TCG​TCC​TGT​CT	120	NM_001001889.1
CAT	CAA​GGT​CTG​GTC​CCA​TAA​A	TGA​CTG​GTA​GTT​GGA​GGT​AA	227	BC051626
SOD	GTC​CGC​ACT​TCA​ACC​CTC​A	TCC​TCA​TTG​CCA​CCC​TTC​C	217	BC055516
GSH-PX	AGA​TGT​CAT​TCC​TGC​ACA​CG	AAG​GAG​AAG​CTT​CCT​CAG​CC	94	AW232474
β-actin	CCG​TGA​CAT​CAA​GGA​GAA​GC	TAC​CGC​AAG​ATT​CCA​TAC​CC	194	AF057040.1

### 2.6 Statistical Analysis

Results were expressed as mean ± standard error of the mean (sem). SPSS 16.0 was used to analyze statistical differences. Then, after testing for normality and homogeneity of variance between groups, the results were subjected to a one-way analysis of variance (ANOVA) followed by Tukey’s test. In addition, RNAi experiments were analyzed by t-test analysis using independent samples. Differences were set at *p* < 0.05.

## 3 Results

### 3.1 Effect of Betaine on the Gene Expression Related to Wnt10b/β-Catenin Signaling in Zebrafish

**FIGURE 1:**
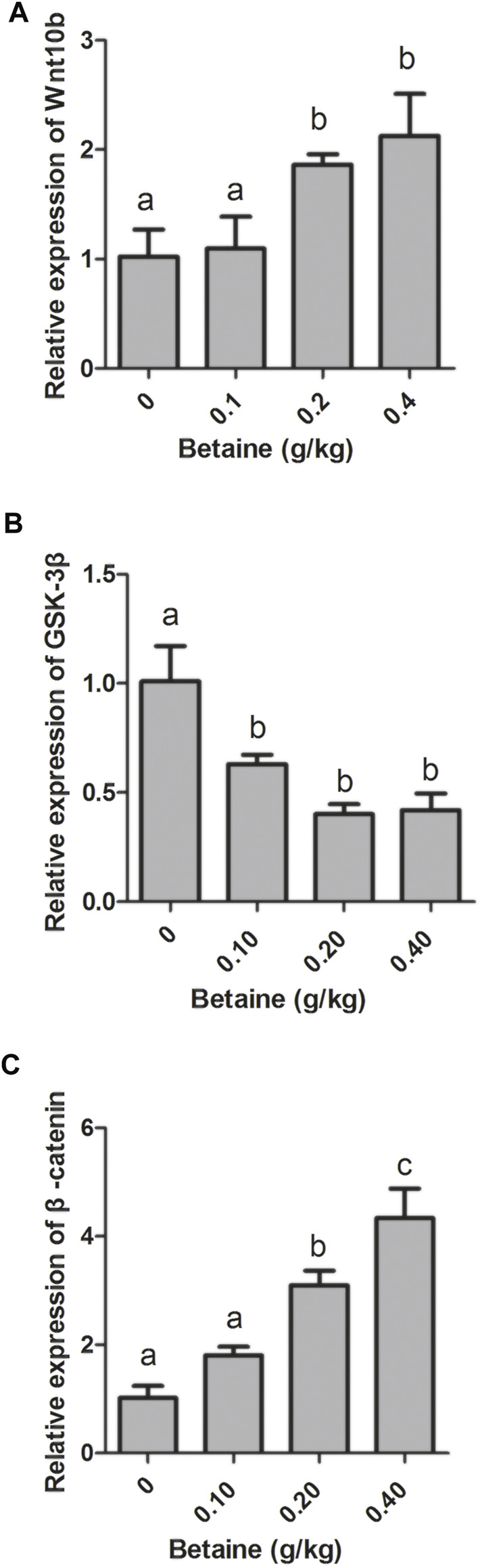
Effect of betaine on the mRNA expression of Wnt10b, GSK-3β, and β-catenin in the liver of zebrafish. **(A)** The mRNA expression of Wnt10b; **(B)** The mRNA expression of GSK-3β; **(C)** The mRNA expression of β-catenin. Values are expressed as means ± sem (*n* = 3). Statistically significant differences are denoted by different letters (*p* < 0.05).

Compared with control, 0.2 and 0.4 g/kg betaine treatments significantly induced Wnt10b gene expression, while 0.1, 0.2 and 0.4 g/kg betaine treatments significantly decreased GSK-3β gene expression (Figures 1A,B). However, the gene expression of GSK-3β was not significantly different between 0.1, 0.2 and 0.4 g/kg betaine treatments (Figure 1B). Furthermore, betaine enrichment of diet at 0.2 and 0.4 g/kg significantly enhanced β-catenin gene expression (Figure 1C).

### 3.2 Effect of Betaine on the Gene Expression of Antioxidant Enzymes in Zebrafish

**FIGURE 2:**
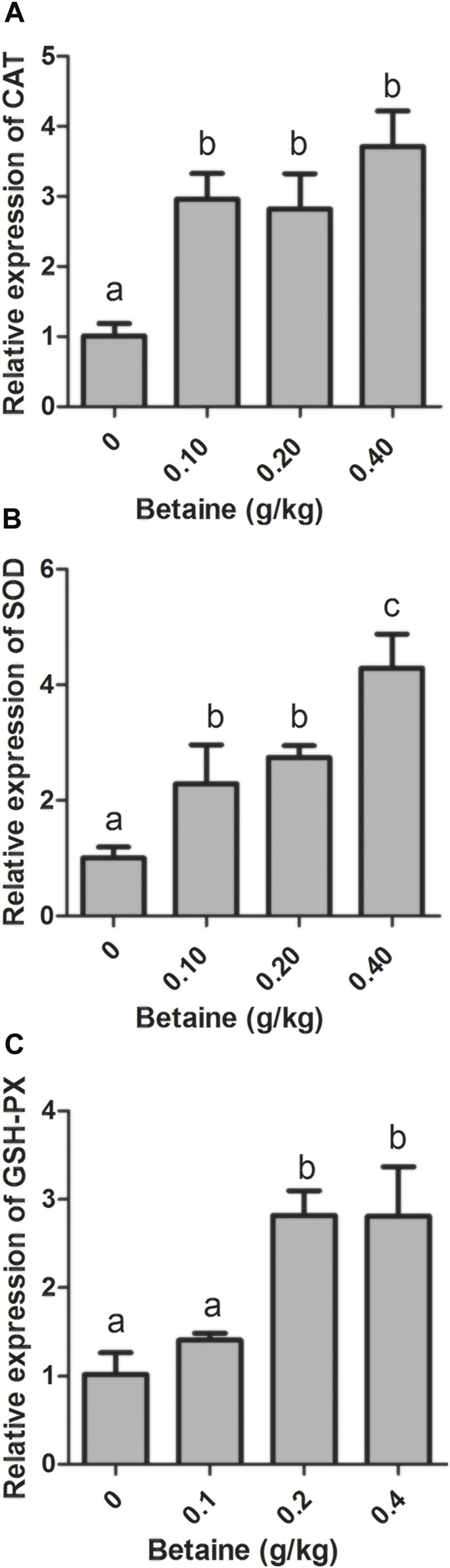
Effect of betaine on the mRNA expression of CAT, SOD, and GSH-PX in the liver of zebrafish. **(A)** The mRNA expression of CAT; **(B)** The mRNA expression of SOD; **(C)** The mRNA expression of GSH-PX. Values are expressed as means ± sem (*n* = 3). Statistically significant differences are denoted by different letters (*p* < 0.05).

0.1, 0.2 and 0.4 g/kg betaine treatments significantly induced CAT and SOD gene expression compared with control (Figures 2A,B). However, there was no significant difference in CAT gene expression between three betaine treatments (Figure 2A). In addition, 0.2 and 0.4 g/kg betaine treatments significantly induced GSH-PX gene expression (Figure 2C).

### 3.3 Effect of Betaine on the Level of Reactive Oxygen Species in Zebrafish

**FIGURE 3:**
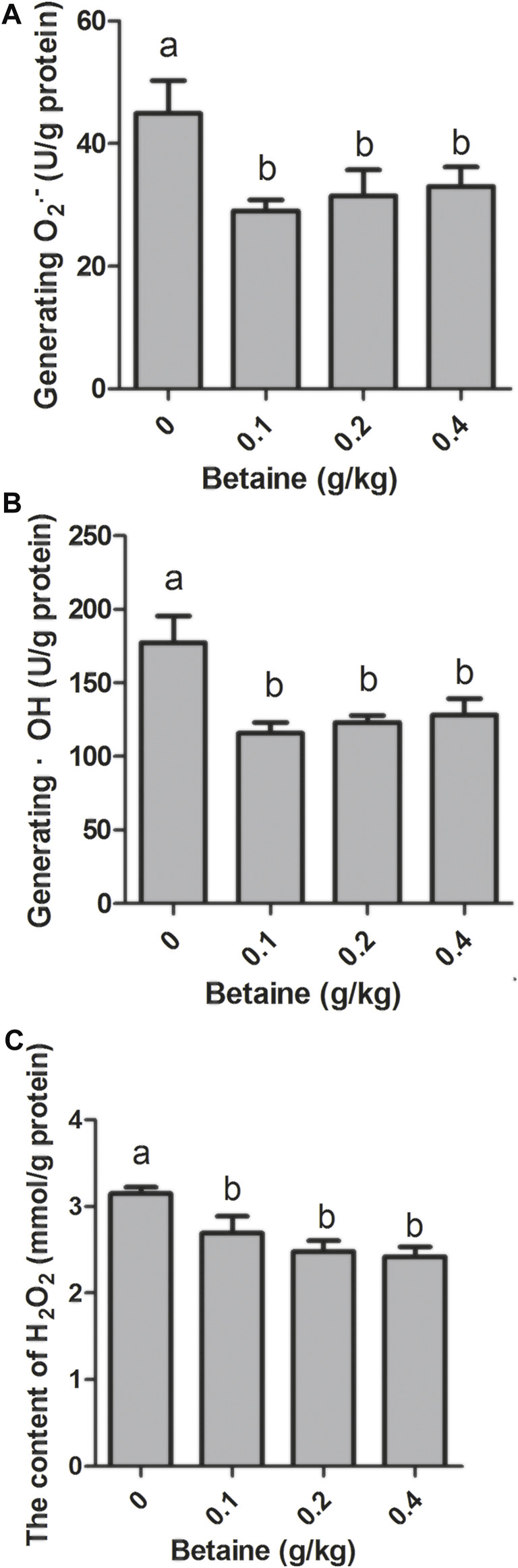
Effect of betaine on the level of ROS in the liver of zebrafish. **(A)** The level of O_2_^·−^; **(B)** The level of ·OH; **(C)** The level of H_2_O_2_. Values are expressed as means ± sem (*n* = 3). Statistically significant differences are denoted by different letters (*p* < 0.05).

0.1, 0.2 and 0.4 g/kg betaine treatments significantly reduced O_2_^·−^ level, but there was no significant difference between 0.1, 0.2 and 0.4 g/kg betaine treatments (Figure 3A). In addition, three betaine treatments significantly reduced the levels of ·OH and H_2_O_2_ (Figures 3B,C).

### 3.4 Effect of Betaine on the Antioxidant Enzyme Activity in Zebrafish

**FIGURE 4:**
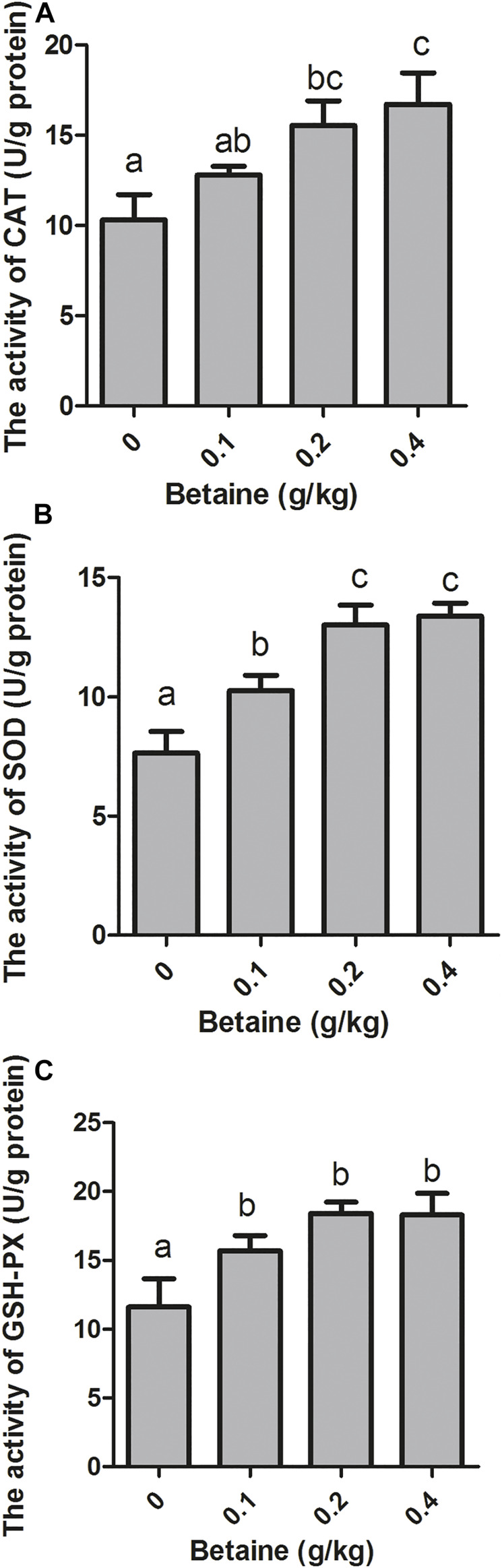
Effect of betaine on the activities of antioxidant enzymes in the liver of zebrafish. **(A)** The activity of CAT; **(B)** The activity of SOD; **(C)** The activity of GSH-PX. Values are expressed as means ± sem (*n* = 3). Statistically significant differences are denoted by different letters (*p* < 0.05).

0.2 and 0.4 g/kg betaine treatments significantly increased CAT activity, while no significant difference was observed between 0.2 and 0.4 g/kg betaine treatments (Figure 4A). Furthermore, betaine enrichment of diet at 0.1, 0.2 and 0.4 g/kg significantly increased the activities of SOD and GSH-PX (Figures 4B,C).

### 3.5 Effect of Wnt10b RNAi on the Gene Expression Related to Wnt10b/β-Catenin Signaling in Zebrafish

Interference of Wnt10b RNA significantly reduced Wnt10b gene expression and significantly increased GSK-3β gene expression (Table 4). In addition, Wnt10b RNAi significantly reduced the expression of β-catenin gene (Table 4).

**TABLE 4:** Effect of Wnt10b RNA interference on the gene expression level in the liver of zebrafish.

Genes	Control group (0.4 g/kg betaine)	RNAi group (Wnt10b RNAi and 0.4 g/kg betaine)	*p* Value
Wnt10b	1.02 ± 0.23	0.42 ± 0.09	<0.05
GSK-3β	1.01 ± 0.19	3.32 ± 0.82	<0.05
β-catenin	1.02 ± 0.25	0.57 ± 0.06	<0.05
CAT	1.03 ± 0.27	0.47 ± 0.05	<0.05
SOD	1.00 ± 0.03	0.51 ± 0.11	<0.05
GSH-PX	1.01 ± 0.13	0.48 ± 0.03	<0.05

Values are expressed as means ± sem (*n* = 3). Statistically significant differences are denoted by *p* values.

### 3.6 Effect of Wnt10b RNAi on the Gene Expression of Antioxidant Enzymes in Zebrafish

Interference with Wnt10b RNA significantly reduced the gene expression of CAT and SOD compared to controls (Table 4). In addition, Wnt10b RNAi in zebrafish liver also significantly suppressed GSH-PX gene expression (Table 4).

### 3.7 Effect of Wnt10b RNAi on the Level of Reactive Oxygen Species and Activity of Antioxidant Enzymes in Zebrafish

Wnt10b RNAi treatment significantly increased the level of O_2_^·−^ (Table 5). Interference of Wnt10b RNA also significantly increased the levels of ·OH and H_2_O_2_ (Table 5). Furthermore, Wnt10b RNAi treatment significantly reduced SOD, CAT and GSH-PX activities in zebrafish liver (Table 5).

**TABLE 5:** Effect of Wnt10b RNA interference on the level of ROS and activities of antioxidant enzymes in the liver of zebrafish.

ROS level and antioxidant enzyme activity	Control group (0.4 g/kg betaine)	RNAi group (Wnt10b RNAi and 0.4 g/kg betaine)	*p* Value
O_2_^·−^ level (U/g protein)	54.30 ± 3.73	64.63 ± 4.01	<0.05
·OH level (U/g protein)	132.50 ± 6.21	150.54 ± 4.94	<0.05
H_2_O_2_ level (mmol/g protein)	3.48 ± 0.38	4.50 ± 0.15	<0.05
CAT activity (U/g protein)	10.90 ± 0.40	9.03 ± 0.33	<0.05
SOD activity (U/g protein)	6.85 ± 0.68	5.38 ± 0.39	<0.05
GSH-PX activity (U/g protein)	12.30 ± 1.01	10.27 ± 0.57	<0.05

Values are expressed as means ± sem (*n* = 3). Statistically significant differences are denoted by *p* values.

## 4 Discussion

**FIGURE 5:**
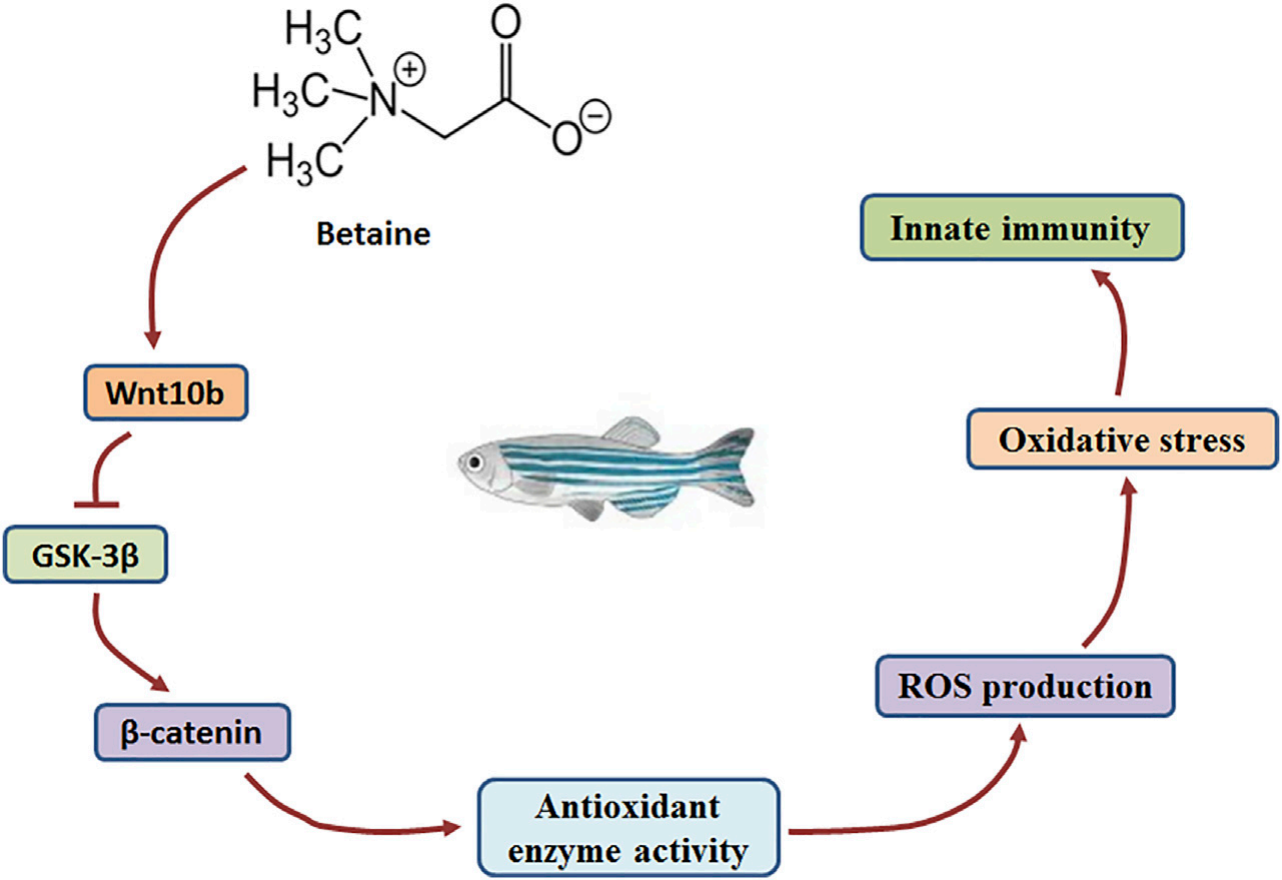
The mechanism by which betaine regulates the production of ROS through Wnt10b/β-catenin signaling pathway in the zebrafish liver.

Wnt molecules play a key role in the Wnt/β-catenin signaling pathway (Cossu and Borello, 1999; Ridgeway, et al., 2000; Taelman, et al., 2010; Valenta, et al., 2012). GSK-3β activity is hindered as Wnt molecules bind to receptors on the cell membrane (Taelman, et al., 2010). In addition, β-catenin levels are closely related to the activity of GSK-3β (Huelsken and Behrens, 2002). In the present study, gene expression of both Wnt10b and β-catenin was induced, but GSK-3β gene expression was decreased by betaine treatment in zebrafish liver. Our findings suggest that betaine can stimulate the Wnt/β-catenin signaling pathway by inducing Wnt10b and inhibiting GSK-3β expression (Figure 5).

Under conditions of oxidative stress, ROS production in fish will be enhanced over time. However, overproduction of ROS can damage cellular components and interfere with cellular metabolism (Lushchak, 2014). Previously, betaine has been shown to have antioxidant properties in various animals. Betaine attenuates oxidative stress in the liver of rat fed with the high-fructose and thioacetamide diet (Doğru-Abbasoğlu, et al., 2018; Heidari, et al., 2018). In addition, betaine reduces the negative effects of heat stress-stimulated oxidative status in broilers (Wen, et al., 2019), and betaine alleviates heat stress in dairy cows (Hall, et al., 2016; Zhang, et al., 2014). In this study, different concentrations of betaine in diet reduced the levels of O_2_^.−^, ·OH and H_2_O_2_ within fish liver. Furthermore, the increasing betaine content in diet significantly induced the gene expression and activity of CAT, SOD and GSH-PX in zebrafish liver. In the current study, we also found that the higher content of betaine can better reduce the level of ROS and increase the activity of antioxidant-related enzymes, which is consistent with the results of previous studies on betaine.

To confirm that Wnt10b plays a key role in regulating ROS levels, fish were intraperitoneally injected with Wnt10b dsRNA and ROS production was further detected in zebrafish liver. The results showed that β-catenin gene expression was inhibited, and the Wnt/β-catenin signaling pathway was inhibited by Wnt10b RNAi. In addition, Wnt10b RNAi increased the levels of O_2_^.−^, ·OH and H_2_O_2_ in zebrafish liver, while decreasing the activities of SOD, CAT and GSH-PX. Wnt/β-catenin signaling pathway was previously found to play a role in regulating oxidative stress in MC3T3-E1 cells (Qi, et al., 2016). Our findings also suggest that the Wnt/β-catenin signaling pathway plays a key role in regulating antioxidant-related enzyme activities and ROS levels. Therefore, for betaine-induced Wnt/β-catenin signaling, it clearly demonstrated that betaine can inhibit ROS production in zebrafish liver through the Wnt/β-catenin signaling pathway (Figure 5).

In addition, betaine has three active methyl groups and plays a unique role in animal nutrition metabolism. Methyl groups are necessary for animal metabolism and cannot be synthesized by animals themselves. Therefore, betaine is one of the most efficient active methyl donors due to its unique structure (Kidd, et al., 1997). It has been previously found that if animals have enough betaine in their bodies, they can store more methionine for other metabolic activities (Lipiński, et al., 2012; Pereira, et al., 2018). Betaine is involved in regulating various physiological activities of animals (Zhang, et al., 2016). In addition, betaine has the ability to modulate osmotic changes and water imbalance during heat stress (Mahmoudnia and Madani, 2012; Zhou, et al., 2012). In the poultry industry, betaine can maintain the moisture of poultry cells and improve the ability of poultry to resist heat stress (Saeed, et al., 2017). In the current study, we observed that betaine reduced ROS levels and increased the activities of antioxidant-related enzymes. The ability of betaine to reduce ROS levels may also be related to osmoprotectant and methyl-donating properties. However, the specific mechanism needs to be further studied in future research.

## 5 Conclusion

In conclusion, a mechanism by which betaine modulates oxidative stress via Wnt10b/β-catenin signaling was discovered in zebrafish liver. Our findings suggest that betaine can induce Wnt10b/β-catenin signaling in zebrafish liver. ROS levels are reduced in zebrafish liver, but betaine enhances the activity of antioxidant-related enzymes. Furthermore, the results of Wnt10b RNAi indicated that the Wnt10b/β-catenin signaling pathway plays a key role in regulating ROS production and antioxidant-related enzymatic activities. Taken together, betaine can inhibit ROS production in zebrafish liver through Wnt10b/β-catenin signaling pathway.

## Data Availability

The original contributions presented in the study are included in the article/Supplementary Material, further inquiries can be directed to the corresponding authors.
